# Supplementary figures and images for: Comparison of three clustering approaches for detecting novel environmental microbial diversity
Source: PeerJ. 2016 Feb 25;4:e1692. doi: 10.7717/peerj.1692 (PMC4782723; doi:10.7717/peerj.1692)

Sequence similarity [%] to closest hit in PR<sup>2</sup>

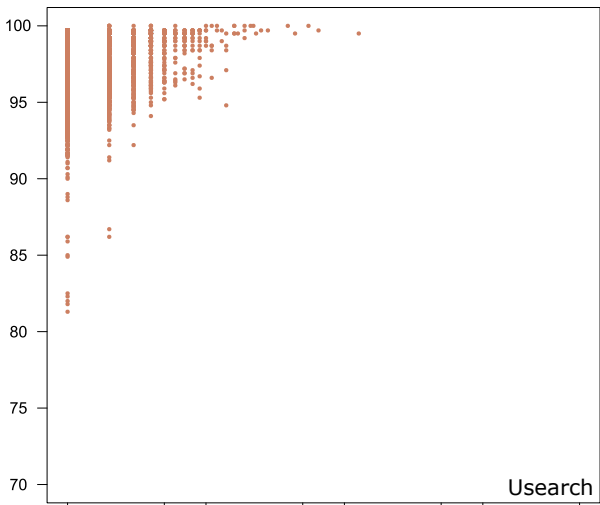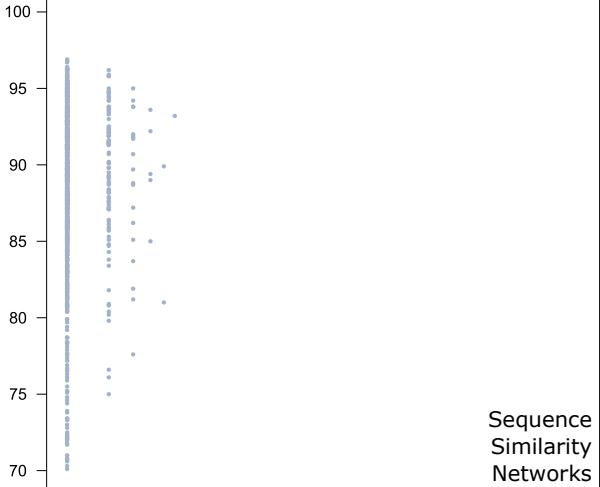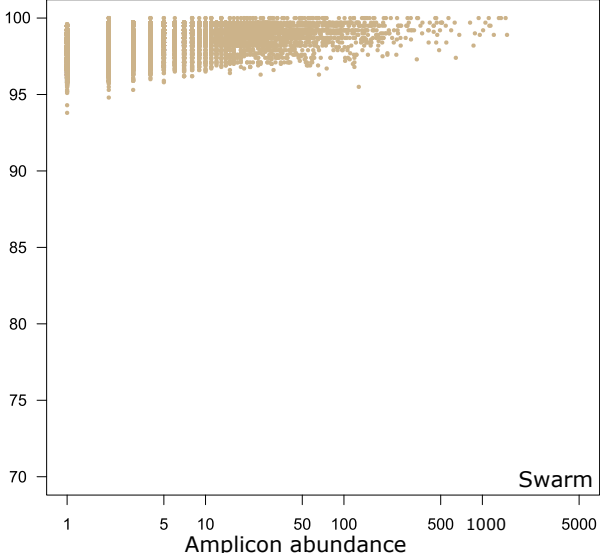

Supplement: Figure S1 — The figure represents a subset of the data shown in Fig. 2. Instead of showing all points, we specifically highlighted the fraction of amplicons which were clustered into exclusively environmental OTUs by exclusively one approach. [file peerj-04-1692-s002.pdf]
