# Supplementary material for: Comparison of three clustering approaches for detecting novel environmental microbial diversity
Source: PeerJ. 2016 Feb 25;4:e1692. doi: 10.7717/peerj.1692 (PMC4782723; doi:10.7717/peerj.1692)
Supplement: File S1 — File describing the commands used to run the analyses described in the manuscript [file peerj-04-1692-s003.zip › Supplementary_File_1.html]

xml version="1.0" encoding="utf-8"?


Comparison of three clustering approaches for detecting novel environmental microbial diversity


# Comparison of three clustering approaches for detecting novel environmental microbial diversity

Dominik Forster, Micah Dunthorn, Thorsten Stoeck, Frédéric Mahé

Supplementary File 1

## Table of Contents

- 1 Disclaimer
- 2 BioMarKs data
- 3 Reference sequences
- 4 Taxonomic assignments
- 5 Clustering and Similarity-based Network analysis 
  - 5.1 Usearch
  - 5.2 Network analysis
  - 5.3 Swarm

## 1 Disclaimer

The purpose of this document is too provide the reader with details on
the bioinformatics methods used to prepare the paper "Comparison of
three clustering approaches for detecting novel environmental
microbial diversity". The code snippets and shell commands presented
here were executed on a Debian GNU/Linux 8, and might have to be
adapted to your particular system. Use them carefully.

## 2 BioMarKs data

The sample design and sample processing, as well as Roche/454 GS FLX
Titanium sequencing of the V4 region of 18S rDNA, is detailed in
Massana et al. (2015). Raw reads were quality filtered and checked for
chimeras with both UCHIME (Edgar, 2010) and ChimeraSlayer (Haas et
al., 2011). The 1,476,249 cleaned V4 DNA and RNA reads were
dereplicated into 312,503 strictly identical amplicons using a custom
bash script. That script can now be entirely replaced with a single
command of vsearch v1.4.

```
INPUT="raw.fasta"
OUTPUT="biomarks_v4.fas"
VSEARCH="/usr/local/bin/vsearch"

"${VSEARCH}" \
    --derep_fulllength "${INPUT}" \
    --sizein \
    --sizeout \
    --fasta_width 0 \
    --relabel_md5 \
    --output "${OUTPUT}"
```

## 3 Reference sequences

For reference amplicons, we used PR2 v203 taxonomic reference database
(Guillou et al., 2012). From this database we extracted 115,043
taxonomically identified V4 amplicons.

```
REF="PR2_network_IDs.fasta"
ENV="biomarks_v4.fas"
FASTA="biomarks_v4_plus_PR2.fas"

cp "${ENV}" "${FASTA}"

# Add a fake abundance value to reference sequences, mark and remove
# sequences with ambiguous nucleotides, convert to lowercase, append
# to environmental sequences
sed -e '/^>/ s/$/_1/' \
    -e '/^>/ ! s/[^ACGT]/@/g' \
    -e '/^>/ ! y/ACGT/acgt/' "${REF}" | \
    paste - - | \
    grep -v "@" | \
    tr "\t" "\n" >> "${FASTA}"
```

Swarm requires sequences without ambiguous nucleotides. We needed to
exclude 6,022 references sequences containing ambiguous
nucleotides. The remaining 109,021 reference sequences will be
combined with the environmental amplicons in swarm analyses.

## 4 Taxonomic assignments

To compare the novel diversity uncovered by each clustering approach,
we analyzed OTUs consisting of exclusively environmental
amplicons. For each amplicon in exclusively environmental OTUs, we
conducted global pairwise alignments of these amplicons with all PR2
references in separate VSEARCH (using options `-allpairs_global`,
`-iddef 1` and `-id 0.70`) analyses, and gathered the highest global
alignment score in % similarity to any reference sequence. This
revealed how divergent the novel diversity of each clustering approach
was with regard to taxonomically identified references. We also
compared if the same environmental amplicons were classified as novel
diversity among the different approaches.

```
DATASET="biomarks_v4.fas"
REFERENCES="PR2_network_IDs.fasta"
TABLE="biomarks_v4.results"
VSEARCH="/usr/local/bin/vsearch"

"${VSEARCH}" \
    --usearch_global "${DATASET}" \
    --db "${REFERENCES}" \
    --userout "${TABLE}" \
    --userfields query+target+id1 \
    --iddef 1 \
    --id 0.70
```

## 5 Clustering and Similarity-based Network analysis

Three de novo clustering approaches were used to cluster the combined
amplicons.

### 5.1 Usearch

First, usearch v8.0.1623 (Edgar, 2010), with a 97% global similarity
value using options `-cluster_smallmem` and `-sortedby size`.

```
DATASET="biomarks_v4.fas"
REFERENCES="PR2_network_IDs.fasta"
FASTA="biomarks_v4_plus_PR2.fas"
USEARCH="/usr/local/bin/usearch8.0.1623_i86linux32"
UC="biomarks_v4_plus_PR2_usearch8_97.uc"

# Decompress environmental sequences
sed -e '/^>/ s/_/;size=/' \
    -e '/^>/ ! y/acgt/ACGT/' "${DATASET}" > "${FASTA}"

# Add PR2 reference sequences
sed -e '/^>/ s/_/;size=/' \
    -e '/^>/ ! y/acgt/ACGT/' \
    -e '/^>PR2/ s/;size=/_/' \
    -e '/^>PR2/ s/_1$/;size=1/' "${REFERENCES}" >> "${FASTA}"

"${USEARCH}" -cluster_smallmem "${FASTA}" -uc "${UC}" -id 0.97 -sortedby size
```

### 5.2 Network analysis

Network topology information was gathered by running a global pairwise
alignment analysis in vsearch v1.1.3 using options `-allpairs_global`
and `-iddef 1`.

```
FASTA="biomarks_v4_plus_PR2.fas"
TABLE="network_input"
VSEARCH="/usr/local/bin/vsearch"

"${VSEARCH}" --allpairs_global "${FASTA}" \
             --userout "${TABLE}" \
             --userfields query+target+id1 \
             --iddef 1 \
             --id 0.97
```

The resulting matrix contained 682,621,198 edges with a weight of at
least 97% global similarity value. Based on this matrix we created
sequence similarity networks in R version 3.2.1 (http://r-project.org)
using "igraph" scripts (Csardi and Nepusz, 2006).

```
############################################################################
####### This script allows to evaluate the composition of connected ########
####### components in RStudio (using iGraph). The Input file should ########
####### be in the format: "Node1 Node2 %ID". The script also allows ########
####### to calculate assortativity values between BioMarKs and PR2. ########
############################################################################

# Load igraph package
library("igraph", lib.loc="~/R/x86_64-unknown-linux-gnu-library/3.1")

# Load network_input file created in bash into RStudio
network_input <- read.table("network_input", head=F)

# Build basic network
network <- graph.data.frame(network_input, directed=F)

# Build network on several sequence similarity levels by removing
# edges below the chosen %ID (default is 85% due to the VSEARCH query
# in bash). For examples we use some of the most commonly used
# thresholds (97%, 95%, 90%)
network_97 <- delete.edges(network, which(E(network)$V3<97))
network_95 <- delete.edges(network, which(E(network)$V3<95))
network_90 <- delete.edges(network, which(E(network)$V3<90))

# Partition the network(s) into CCs, keep only CCs which contain at
# least 3 nodes
network_decomposed <- decompose.graph(network, min.vertices=3)
network_97_decomposed <- decompose.graph(network_97, min.vertices=3)
network_95_decomposed <- decompose.graph(network_95, min.vertices=3)
network_90_decomposed <- decompose.graph(network_90, min.vertices=3)

# Load a new function, which will unveil the CC composition by
# searching for PR2 nodes (previously marked with "^PR2_"). The
# function will count the total number of nodes, the number of
# BioMarKs nodes and the number of PR2 nodes for each CC.
CC_composition <- function(cc){
    n=V(cc)$name
    REFseq=grep("^PR2_", n, value=T, invert=F)
    BIOMseq=grep("^PR2_", n, value=T, invert=T)
    list_elements=list(n, BIOMseq, REFseq)
    detailed_cc=sapply(list_elements, length)
}

# Apply the function on the decomposed network(s) and save the results
# as a data frame for further evaluation (in the following you can
# export the data frames via write.csv() or do more statistical tests
# (e.g. sum) in RStudio).
cc_85_composition <- t(as.data.frame(lapply(network_decomposed, CC_composition)))
cc_90_composition <- t(as.data.frame(lapply(network_90_decomposed, CC_composition)))
cc_95_composition <- t(as.data.frame(lapply(network_95_decomposed, CC_composition)))
cc_97_composition <- t(as.data.frame(lapply(network_97_decomposed, CC_composition)))
write.csv2(cc_85_composition, "cc_85_composition.csv")
write.csv2(cc_90_composition, "cc_90_composition.csv")
write.csv2(cc_95_composition, "cc_95_composition.csv")
write.csv2(cc_97_composition, "cc_97_composition.csv")

# Load a new function, which will help to calculate Assortativity values for
# groups of nodes (e.g. for PR2 reference nodes). The function was originally
# written by Slim Karkar from Eric Bapteste's lab in Paris.
list_assortativity <- function(g,list1) {
    names=V(g)$name
    types=(names %in% list1)*1+1
    if(length(V(g))==sum(types==1)) {anc="NULL"}
    else{
        anc= assortativity.nominal(g,types,directed=F)}
    anc
}

# Before running the function we need to save all PR2 sequence IDs in
# an object.
PR2_IDs <- grep("^PR2_", (V(network)$name), value=T)

# Same for BioMarKs sequence IDs.
BioMarKs_IDs <- grep("^PR2_", (V(network)$name), value=T, invert=T)

# Apply the function on the decomposed network(s) for both ID
# lists. Results can also be exported to csv file using write.csv2().
PR2_ass_85 <- lapply(network_decomposed, list_assortativity, PR2_IDs)
BioMarKs_ass_85 <- lapply(network_decomposed, list_assortativity, BioMarKs_IDs)
PR2_ass_90 <- lapply(network_90_decomposed, list_assortativity, PR2_IDs)
BioMarKs_ass_90 <- lapply(network_90_decomposed, list_assortativity, BioMarKs_IDs)
PR2_ass_95 <- lapply(network_95_decomposed, list_assortativity, PR2_IDs)
BioMarKs_ass_95 <- lapply(network_95_decomposed, list_assortativity, BioMarKs_IDs)
PR2_ass_97 <- lapply(network_97_decomposed, list_assortativity, PR2_IDs)
BioMarKs_ass_97 <- lapply(network_97_decomposed, list_assortativity, BioMarKs_IDs)
```

### 5.3 Swarm

Third, swarm v2.1.1 (Mahé et al., 2014, 2015), with `-d 1` and `-f`.

```
SWARM="/usr/local/bin/swarm"
THREADS=8
FASTA="biomarks_v4_plus_PR2.fas"
REPRESENTATIVES="${FASTA/.fas/_1f_representatives.fas}"
SWARMS="${FASTA/.fas/_1f.swarms}"
STATS="${FASTA/.fas/_1f.stats}"
STRUCT="${FASTA/.fas/_1f.struct}"
LOG="${FASTA/.fas/_1f.log}"

"${SWARM}" \
    -d 1 -f -t "${THREADS}" \
    -w "${REPRESENTATIVES}" \
    -o "${SWARMS}" \
    -s "${STATS}" \
    -i "${STRUCT}" \
    -l "${LOG}" "${FASTA}"
```

**produce stats**

```
FASTA="biomarks_v4_plus_PR2.fas"
SWARMS="${FASTA/.fas/_1f.swarms}"
STAMPA="biomarks_v4.results"
while read l ; do
    TOTAL=$(tr " " "\n" <<< ${l} | wc -l)
    ENVs=$(tr " " "\n" <<< ${l} | grep -c -v "^PR2_")
    REFs=$(tr " " "\n" <<< ${l} | grep -c "^PR2_")
    SEED=$(cut -d " " -f 1 <<< ${l})
    SEED="${SEED/_*/}"
    TAXO=$(grep -m 1 "^${SEED}" "${STAMPA}" | cut -f 2-)
    echo -e "${SEED}\t${TOTAL}\t${ENVs}\t${REFs}\t${TAXO}"
done < "${SWARMS}" > "${FASTA/.fas/_1f_swarms.csv}"
```

Singleton and doubleton OTUs were excluded from the results of all
three clustering approaches for downstream analyses.

Author: Frédéric Mahé
<mahe@rhrk.uni-kl.de>

Date: [2015-09-27 dim.]

HTML generated by org-mode 6.33x in emacs 23
